# Ion Channel Gene Expression in Lung Adenocarcinoma: Potential Role in Prognosis and Diagnosis

**DOI:** 10.1371/journal.pone.0086569

**Published:** 2014-01-23

**Authors:** Jae-Hong Ko, Wanjun Gu, Inja Lim, Hyoweon Bang, Eun A. Ko, Tong Zhou

**Affiliations:** 1 Department of Physiology, College of Medicine, Chung-Ang University, Seoul, South Korea; 2 Research Center for Learning Sciences, Southeast University, Nanjing, Jiangsu, China; 3 Department of Medicine, University of California San Francisco, San Francisco, California, United States of America; 4 Department of Medicine, University of Arizona, Tucson, Arizona, United States of America; National Taiwan University, Taiwan

## Abstract

Ion channels are known to regulate cancer processes at all stages. The roles of ion channels in cancer pathology are extremely diverse. We systematically analyzed the expression patterns of ion channel genes in lung adenocarcinoma. First, we compared the expression of ion channel genes between normal and tumor tissues in patients with lung adenocarcinoma. Thirty-seven ion channel genes were identified as being differentially expressed between the two groups. Next, we investigated the prognostic power of ion channel genes in lung adenocarcinoma. We assigned a risk score to each lung adenocarcinoma patient based on the expression of the differentially expressed ion channel genes. We demonstrated that the risk score effectively predicted overall survival and recurrence-free survival in lung adenocarcinoma. We also found that the risk scores for ever-smokers were higher than those for never-smokers. Multivariate analysis indicated that the risk score was a significant prognostic factor for survival, which is independent of patient age, gender, stage, smoking history, Myc level, and *EGFR*/*KRAS*/*ALK* gene mutation status. Finally, we investigated the difference in ion channel gene expression between the two major subtypes of non-small cell lung cancer: adenocarcinoma and squamous-cell carcinoma. Thirty ion channel genes were identified as being differentially expressed between the two groups. We suggest that ion channel gene expression can be used to improve the subtype classification in non-small cell lung cancer at the molecular level. The findings in this study have been validated in several independent lung cancer cohorts.

## Introduction

Worldwide, lung cancer is the leading cancer killer, and the overall 5-year survival is only 15% [1]. Approximately 98% of lung cancers are carcinomas that arise from epithelial cells [2]. Lung carcinomas are generally categorized into non-small cell and small cell lung cancers by the size and appearance of the malignant cells. About 80% of lung cancers are non-small cell cancers, and of these, roughly 50% are adenocarcinomas [2], which usually originate in peripheral lung tissue. Lung adenocarcinoma is strongly associated with smoking [3], which has become the most common major type of lung cancer in smokers compared to squamous cell carcinoma. However, adenocarcinoma is also the type of lung cancer most commonly seen in non-smokers and women [4], [5].

At the molecular level, a large number of genes have been found to be involved in lung cancer, such as EGFR signaling pathway genes, tumor suppressor genes, and cell immortalization genes [6]. During the last few years, a pivotal role for ion channel involvement in cancer has emerged [7], [8], [9], [10]. Ion channels are expressed in virtually all living cells and create a pathway for charged ions created from dissolved salts, including calcium (Ca^2+^), potassium (K^+^), sodium (Na^+^), and chloride (Cl^−^) ions, to pass through the lipid membrane. Ion channels are thought to aid cancer by regulating the cell cycle of proliferating cells, interfering with membrane potential, preventing apoptosis, altering intracellular Ca^2+^ balance, and adjusting cell shrinkage [9], [10]. There is also mounting evidence for the active involvement of ion channels in lung cancer pathology. For example, increased expression has been observed for voltage-gated Na^+^ channels [11]; voltage-gated Ca^2+^ channels were found to be involved in the pro-proliferative action of mitogen on a human lung adenocarcinoma cell line [12]; two-P K^+^ channels, one of which was *KCNK9*, were found to be over-expressed in more than 35% of lung tumors [13], and the over-expression of *KCNK9* in cell lines promoted tumor formation and conferred resistance to hypoxia and serum deprivation [14]. In addition, ligand-gated ion channels were also found to be implicated in lung neoplastic progression. For example, nicotinic acetylcholine receptor, a type of ionotropic receptor, regulates cell proliferation, apoptosis, and angiogenesis in lung cancers [15], [16], [17], [18]. Although we have already seen an expansion of the increasing list of ion channels implicated in lung cancer development, the exact role and interplay between ion channels and lung cancer remain controversial. Some contradictory opinions exist in regard to the relationship between ion channels and lung cancer.

In this study, we systematically analyzed the ion channel gene expression pattern in lung adenocarcinoma. We pointed out the potential role of ion channel genes in prognosis and diagnosis in lung adenocarcinoma. We first compared the expression of ion channel genes between normal and tumor tissues in patients with lung adenocarcinoma. Thirty-seven ion channel genes were identified as being differentially expressed between the two groups. Next, we investigated the prognostic power of ion channel genes in lung adenocarcinoma. We assigned a risk score to each lung adenocarcinoma patient based on the expression of 37 differentially expressed ion channel genes. We demonstrated that the risk score effectively predicts overall survival and recurrence-free survival in lung adenocarcinoma. Finally, we investigated the difference in ion channel gene expression patterns between the two major subtypes of non-small cell lung cancer: adenocarcinoma and squamous-cell carcinoma. Thirty ion channel genes were identified as being differentially expressed between the two groups. We suggested that ion channel gene expression can be used to improve the classification of non-small cell lung cancer. All of our results were validated in independent lung cancer cohorts.

## Materials and Methods

### Gene Expression Data

Six independent microarray lung cancer datasets from Japan (JPN) [19], Korea (KOR) [20], Sweden (SWE) [21], Taiwan (TWN) [22], and the United States (USA1 [23] and USA2 [24]), were obtained from the Gene Expression Omnibus (GEO) database for use in this study (Table S1). These datasets were chosen based on the large number of samples. The TWN cohort was used to identify the differentially expressed ion channel genes between normal and tumor tissues. The SWE cohort was used to identify the differentially expressed ion channel genes between lung adenocarcinoma and squamous-cell carcinoma. The other four datasets were used as validation cohorts.

Expression data of paired normal and tumor tissues from adenocarcinoma patients were available in the TWN and USA1 cohorts. The SWE, USA2, and KOR cohorts consisted of both lung adenocarcinoma and squamous-cell carcinoma patients. Information on overall survival was available for the USA2 and JPN cohorts. Information on recurrence-free survival was available in the KOR and JPN cohorts.

### Microarray Data Preprocessing

The GC robust multichip average (GCRMA) algorithm [25] in Bioconductor was used to normalize the expression level of each probe set for the microarray data. Only the probe sets present (determined by function “mas5calls” in the Bioconductor “affy” package) in at least two-third of the samples were retained. We further limited our analysis to the probe sets with unique annotations. The genes on chromosomes X and Y were excluded to avoid the potentially confounding gender factor.

### Ion Channel Genes

The definition of human ion channel genes was obtained from GeneCards [26], [27]. In total, we collected 280 ion channels, including voltage-dependent and non-voltage-dependent ion channels (Table S2).

### Statistical Analysis

For the TWN cohort, a paired t-test was used to identify the genes that were differentially expressed between normal and tumor tissues. For the SWE cohort, genes differentially expressed between lung adenocarcinoma and squamous-cell carcinoma were detected by two-tailed t-test. The *P*-values were adjusted by Benjamini & Hochberg correction [28]. We didn’t use Bonferroni correction here because we didn’t want to overlook the genes with real but moderate differences. Although Bonferroni correction controls the family-wise error rate efficiently, it may lead to a very high rate of false negatives. Based on the expression of the genes differentially expressed between normal and tumor tissues, we assigned a risk score to each patient. The risk score was calculated using a linear combination of weighted gene expression as shown below:
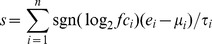



Here, *s* is the risk score of the patient; *n* is the number of differentially expressed genes; *fc_i_* denotes the fold change in expression between normal and tumor tissues (tumor/normal) for the *i* ^th^ gene, which is derived from the discovery cohort (TWN); “sgn” denotes the sign function; *e_i_* denotes the expression level of gene *i*; and *µ_i_* and *τ_i_* are the mean and standard deviation of the gene expression values for gene *i* across all samples, respectively. “sgn(log_2_ *fc_i_*)” is the weight for each gene. We hypothesized that one gene could be regarded as a poor survival related gene if its expression value is higher in lung tumor tissues in comparison with normal tissues. If one ion channel gene is up-regulated in tumor tissue, the weight “sgn(log_2_ *fc_i_*)” for this gene will be 1. On the contrary, if the ion channel gene is down-regulated in tumor tissue, the weight for this gene will be –1. Therefore, according to the risk score formula, patients with higher expression in the up-regulated genes and lower expression in the down-regulated genes tend to have higher risk scores and higher risk scores imply worse outcome in a monotone fashion. Patients were divided into high-score and low-score groups, with the median of the risk score as the threshold value. The median of the risk score was approximately equal to zero in each cohort (Figure S1).

All the statistical analyses were conducted by the R platform. Kaplan-Meier survival analysis was used to measure the fraction of patients surviving for a certain amount of time. The statistical significance between two Kaplan-Meier curves was determined by log-rank test using the “survdiff” function in the “survival” library. Cox model was used to obtain the hazard ratio, which analyzed the effect of one or several risk factors on survival. Both univariate and multivariate Cox proportional hazards regression was conducted by the “coxph” function. Principal component analysis (PCA) was used in this study to visualize the variance in high-dimensional gene expression data. We applied the “dudi.pca” function in the “ade4” library to conduct PCA. Hierarchical clustering was performed to detect and visualize the genes with similar expression patterns. The corresponding gene expression heatmaps were generated by the “heatmap.2” function in the “gplots” library with Ward’s method and Manhattan distance.

## Results

### Differentially Expressed Ion Channel Genes between Normal and Tumor Tissues in Lung Adenocarcinoma

**Figure 1:**
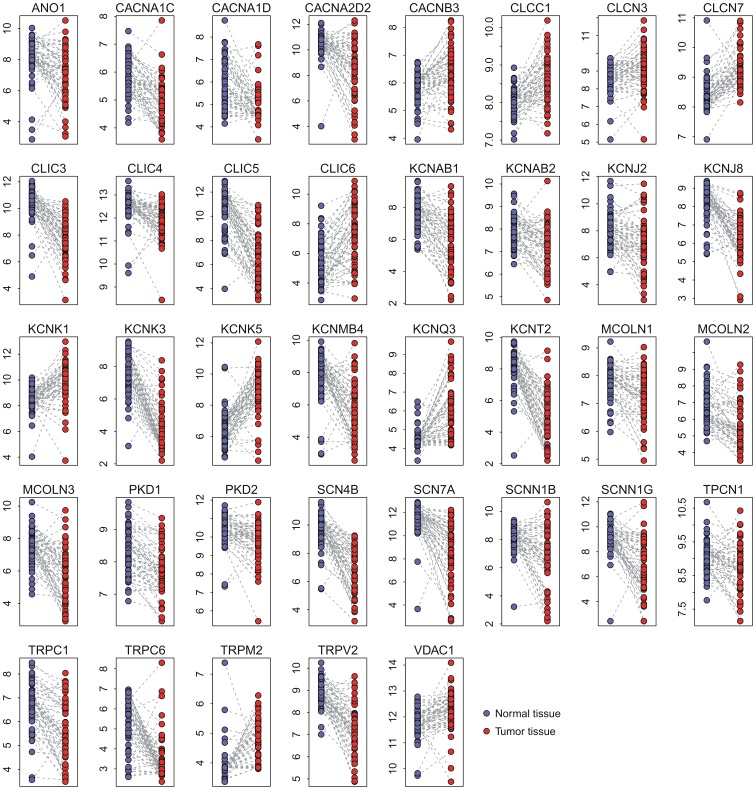
The ion channel genes differentially expressed between normal and tumor tissues in the TWN cohort. Paired normal and tumor tissues from 60 lung adenocarcinoma patients were included in the comparison. In total, 37 ion channel genes were identified as dysregulated. Y-axis: log2-transformed expression values.

We first explored the difference in gene expression between normal and tumor tissues in lung adenocarcinoma in the TWN cohort. Paired normal and tumor tissues from 56 adenocarcinoma patients were included. Paired t-test was used to detect the differentially expressed genes between the normal and tumor tissues. In total, 37 ion channel genes were identified as being differentially expressed between the two groups (adjusted *P*<0.001) (Table 1). Ten ion channel genes were up-regulated in tumor tissues, while 27 ion channel genes were down-regulated (Figure 1).

**Table 1:** Comparison in gene expression level between normal and tumor tissues.

Gene symbol	Gene title	Fold change^a^	Adjuste *P*-value^b^
*ANO1*	anoctamin 1, calcium activated chloride channel	0.48	6.1×10^−5^
*CACNA1C*	calcium channel, voltage-dependent, L type, alpha 1C subunit	0.56	1.8×10^−9^
*CACNA1D*	calcium channel, voltage-dependent, L type, alpha 1D subunit	0.54	3.3×10^−6^
*CACNA2D2*	calcium channel, voltage-dependent, alpha 2/delta subunit 2	0.27	7.2×10^−7^
*CACNB3*	calcium channel, voltage-dependent, beta 3 subunit	1.48	2.8×10^−5^
*CLCC1*	chloride channel CLIC-like 1	1.52	1.6×10^−8^
*CLCN3*	chloride channel, voltage-sensitive 3	1.39	5.7×10^−4^
*CLCN7*	chloride channel, voltage-sensitive 7	1.80	2.1×10^−12^
*CLIC3*	chloride intracellular channel 3	0.17	6.6×10^−13^
*CLIC4*	chloride intracellular channel 4	0.62	8.1×10^−7^
*CLIC5*	chloride intracellular channel 5	0.06	3.3×10^−20^
*CLIC6*	chloride intracellular channel 6	3.28	3.7×10^−8^
*KCNAB1*	potassium voltage-gated channel, shaker-related subfamily, beta member 1	0.26	2.1×10^−12^
*KCNAB2*	potassium voltage-gated channel, shaker-related subfamily, beta member 2	0.62	1.2×10^−5^
*KCNJ2*	potassium inwardly-rectifying channel, subfamily J, member 2	0.50	4.8×10^−5^
*KCNJ8*	potassium inwardly-rectifying channel, subfamily J, member 8	0.25	2.8×10^−15^
*KCNK1*	potassium channel, subfamily K, member 1	2.32	5.1×10^−6^
*KCNK3*	potassium channel, subfamily K, member 3	0.09	3.2×10^−20^
*KCNK5*	potassium channel, subfamily K, member 5	4.77	1.5×10^−12^
*KCNMB4*	potassium large conductance calcium-activated channel, subfamily M, beta member 4	0.24	4.3×10^−8^
*KCNQ3*	potassium voltage-gated channel, KQT-like subfamily, member 3	2.69	1.4×10^−8^
*KCNT2*	potassium channel, subfamily T, member 2	0.10	2.4×10^−18^
*MCOLN1*	mucolipin 1	0.72	6.2×10^−5^
*MCOLN2*	mucolipin 2	0.54	1.9×10^−5^
*MCOLN3*	mucolipin 3	0.29	6.4×10^−9^
*PKD1*	polycystic kidney disease 1 (autosomal dominant)	0.69	1.6×10^−7^
*PKD2*	polycystic kidney disease 2 (autosomal dominant)	0.64	1.7×10^−5^
*SCN4B*	sodium channel, voltage-gated, type IV, beta subunit	0.13	1.5×10^−14^
*SCN7A*	sodium channel, voltage-gated, type VII, alpha subunit	0.16	4.2×10^−10^
*SCNN1B*	sodium channel, non-voltage-gated 1, beta subunit	0.36	1.3×10^−6^
*SCNN1G*	sodium channel, non-voltage-gated 1, gamma subunit	0.20	6.0×10^−10^
*TPCN1*	two pore segment channel 1	0.76	7.8×10^−4^
*TRPC1*	transient receptor potential cation channel, subfamily C, member 1	0.49	5.3×10^−8^
*TRPC6*	transient receptor potential cation channel, subfamily C, member 6	0.31	4.6×10^−9^
*TRPM2*	transient receptor potential cation channel, subfamily M, member 2	1.53	1.2×10^−5^
*TRPV2*	transient receptor potential cation channel, subfamily V, member 2	0.34	1.2×10^−13^
*VDAC1*	voltage-dependent anion channel 1	1.36	6.1×10^−5^

a Fold change is calculated by dividing the expression of tumor tissue by the expression of normal tissue.

b *P*-value is calculated by paired t-test and adjusted by Benjamini & Hochberg correction.

Three genes (*CACNA1D*, *CACNA2D1*, and *CACNA2D2*) coding for the α subunit of voltage-dependent Ca^2+^ channels were down-regulated in tumor tissues. All the Na^+^ channel genes (*SCN4B*, *SCN7A*, *SCNN1B*, and *SCNN1G*) listed in Table 1, including voltage-gated and non-voltage-gated Na^+^ channels, were down-regulated in tumor tissues. Two genes (*PKD1* and *PKD2*) coding for transient receptor potential polycystic (TRPP), a family of transient receptor potential (TRP) ion channels, were down-regulated in tumor tissues. The three genes (*MCOLN1*, *MCOLN2*, and *MCOLN3*) coding for the mucolipin subfamily of TRP channels were also down-regulated in tumor tissues. However, the expression patterns of K^+^ channel and Cl^−^ channels were more heterogeneous. For example, three chloride intracellular channel genes (*CLIC3*, *CLIC4*, and *CLIC5*) were down-regulated, while *CLIC6* was up-regulated three-fold in tumor tissues.

**Figure 2:**
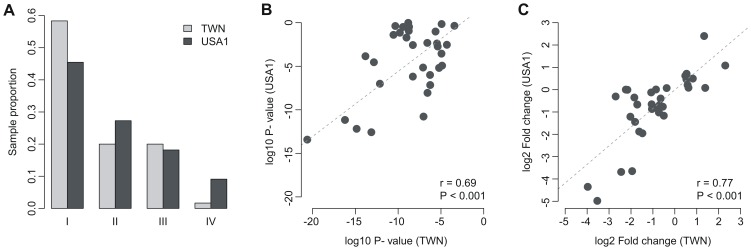
Comparison between the TWN and USA1 cohorts. (A) Comparison of cancer stage distribution between the TWN and USA1 cohorts; (B) Correlation of *P*-value generated by paired t-test (tumor vs. normal tissues) between TWN and USA1 cohort; and (C) Correlation of fold change of gene expression level (tumor vs. normal tissues) between the TWN and USA1 cohorts.

To validate gene expression profiling in another cohort, we accessed another publicly available microarray dataset on lung adenocarcinoma (USA1) where the gene expression data from 33 pairs of normal and tumor tissue from lung were available. We did not find a significant difference (χ^2^ test *P* = 0.352) in the patient stage distribution between the TWN and USA1 cohorts (Figure 2A). However, there was significant difference in patient age, gender, and smoking history between the two cohorts (Table S3).

Four of the 37 dysregulated ion channel genes in the TWN cohort were absent in the USA1 dataset due to a platform difference. Therefore, we validated the remaining 33 genes in the USA1 cohort. Paired t-test indicated that 23 of the 33 genes were also differentially expressed (adjusted *P*<0.05) between normal and tumor tissues in the USA1 cohort (Table S4 and Figure S2). Significant positive correlation in log10-transformed *P*-value (generated by paired t-test) was observed between the TWN and USA1 cohorts (Figure 2B). The direction of differential expression in the TWN cohort was reproduced in the USA1 cohort. Fold change of expression level in the TWN cohort was also strongly correlated with that in the USA1 cohort (Figure 2C).

### Expression of Ion Channel Genes Predicting Overall Survival in Lung Adenocarcinoma

**Figure 3:**
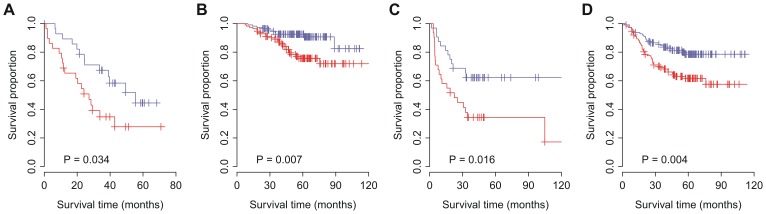
Kaplan-Meier curves for the patients with lung adenocarcinoma. The expression of iLAS predicts poor clinical outcome in lung adenocarcinoma. Red curves are for the iLAS positive patients while blue curves are for the iLAS negative patients. iLAS positive patients were defined as those having a iLAS risk score greater than the group median score. *P*-values were calculated by log-rank tests for the differences in survival between the iLAS positive and negative groups. (A) iLAS predicts overall survival in the USA2 cohort; (B) iLAS predicts overall survival in the JPN cohort; (C) iLAS predicts recurrence-free survival in the KOR cohort; and (D) iLAS predicts recurrence-free survival in the JPN cohort.

We hypothesized that the 37 ion channel genes differentially expressed between normal and tumor tissues would be predictive of tumor outcome in patients with lung adenocarcinoma. We designated these ion channel genes as the ion channel-based Lung Adenocarcinoma gene Signature (iLAS). Cox proportional hazards regression was conducted for each gene in iLAS. A significant positive correlation (*P*<0.05) was identified between fold change (tumor/normal) and Cox regression coefficient for each dataset except the KOR cohort (*P* = 0.065) (Figure S3). So we assumed that one gene could be regarded as a poor survival related gene if its fold change was smaller than one. We constructed a risk scoring system that combined gene expression information in the iLAS with the fold change listed in Table 1. iLAS-positive patients were defined as those having a risk score greater than the group median score, and the other patients were assigned as iLAS negative. We tested the ability of the risk score to predict overall survival in two independent lung cancer cohorts (USA2 and JPN). The overall survival information for 58 and 226 lung adenocarcinoma patients is available in the USA2 and JPN cohorts, respectively. Kaplan-Meier survival analysis was used to compare the iLAS positive and negative groups. The iLAS signature identified the patients with poor overall survival in both cohorts (*P*<0.05) (Figures 3A and 3B). The association between iLAS status and overall survival was confirmed by univariate Cox proportional hazards regression of survival. iLAS-positive patients had a 2.13-fold increased risk for death in the USA2 cohort and 2.64-fold increased risk in the JPN cohort (Table 2). Cox regression against continuous iLAS score indicated that one-point increment in the risk score raised the hazard of death by 2% and 4% in the USA2 and JPN cohorts, respectively (Table S5).

**Table 2:** Univariate Cox proportional hazards regression of survival by iLAS status for the lung adenocarcinoma patients.

Gategory	Cohort	Number of patients	Hazard ratio	95% Confidence interval	*P*-value
Overall survival	USA2	58	2.13	(1.04, 4.34)	0.038
	JPN	226	2.64	(1.26, 5.50)	0.010
Recurrence-free survival	KOR	63	2.35	(1.15, 4.80)	0.019
	JPN	226	2.10	(1.26, 3.52)	0.005

### iLAS Predicting Recurrence-free Survival in Lung Adenocarcinoma

One challenge of lung cancer research is to identify the patients who are at higher risk of post-resection recurrence. Here, we tested the power of iLAS in predicting recurrence-free survival in two independent lung cancer cohorts (KOR and JPN). There were 63 and 226 lung adenocarcinoma patients with known recurrence-free survival data in the KOR and JPN cohorts, respectively. Kaplan-Meier survival curves indicated that the iLAS signature was able to identify the patients with higher risk of recurrence in both cohorts (*P*<0.05) (Figures 3C and 3D). The association between iLAS status and recurrence-free survival was confirmed by univariate Cox proportional hazards regression of survival. iLAS-positive patients had a 2.35-fold increased risk for recurrence in the KOR cohort and 2.10-fold increased risk in the JPN cohort (Table 2). Cox regression against continuous iLAS score indicated that one-point increment in the risk score raised the hazard of recurrence by 3% and 4% in the KOR and JPN cohorts, respectively (Table S5).

### iLAS and Smoking History

**Figure 4:**
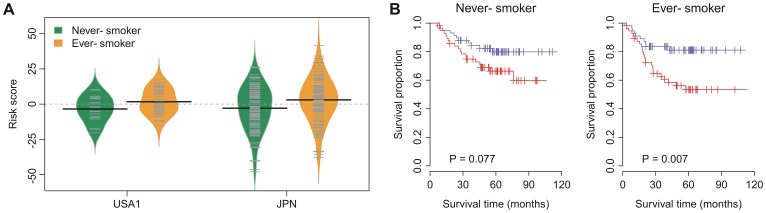
Comparison between patients with and without smoking history. (A) Difference in iLAS risk score between never- and ever- smokers. The iLAS risk scores of ever-smokers are slightly but significantly higher than the scores of never-smokers in the USA1 and JPN cohorts. (B) Kaplan-Meier curves for recurrence-free survival for the patients with and without smoking history. The patients in the JPN cohort were grouped by smoking history. Red curves are for the iLAS positive patients while blue curves are for the iLAS negative patients. iLAS positive patients were defined as those having a iLAS risk score greater than the group median score. *P*-values were calculated by log-rank tests for the differences in survival between the iLAS positive and negative groups.

Cigarette smoking is strongly associated with lung cancers and decreased survival [23]. Therefore, we tested whether iLAS status is affected by the smoking history in the patients with lung adenocarcinoma. The information on smoking history is available in the USA1 and JPN cohorts. There were 19 never-smokers and 39 ever-smokers in the USA1 cohort. In the JPN cohort, there were 115 and 111 lung adenocarcinoma patients with and without a smoking history, respectively. The iLAS risk score for the ever-smokers was slightly but significantly higher than that for the never-smokers in both cohorts (*P* = 0.047 and *P* = 0.004 for the USA1 and JPN cohorts, respectively) (Figure 4A and Table S6). A multivariate Cox regression on recurrence-free survival indicated that iLAS status remained a significant covariate (hazard ratio = 1.43 and *P* = 0.006) in relation to smoking history in the JPN cohort. The iLAS-positive patients had a 1.39-fold (*P* = 0.082) and 1.63-fold (*P* = 0.009) increased risk for recurrence, respectively. Kaplan-Meier survival analysis also demonstrated a significantly reduced survival for iLAS-positive patients in the subset grouped by smoking history (Figure 4B). Taken together, these results suggest that iLAS is associated with clinical outcome and is independent of smoking history.

### Multivariate Analysis with Clinical and Pathological Factors

To investigate the performance of iLAS in comparison with clinical and pathological variables associated with prognosis in lung cancer, a multivariate analysis was conducted in the JPN cohort, the largest dataset in this study. Firstly, clinical factors, such as patient age, gender, stage, and smoking history were included in the multivariate model. Secondly, we took Myc protein level into account. Tumors with increased Myc level have been linked to poor clinical outcomes [29], [30]. Thirdly, the mutation status of oncogenes (*EGFR/KRAS/ALK*) was considered. Mutations in *EGFR*, *KRAS*, and *ALK* are almost always mutually exclusive. A considerable proportion of lung adenocarcinomas develop through acquisition of mutations in *EGFR*, *KRAS*, or *ALK* genes [19], [31], [32]. Multivariate Cox regression of survival indicated that iLAS dichotomized status remained a significant covariate in relation to the clinical and pathological factors in lung adenocarcinoma (Table 3). Cox regression against continuous iLAS score also confirmed that the iLAS signature was associated with poor outcomes and was an independent prognostic factor (Table S7).

**Table 3:** Multivariate Cox proportional hazards regression of survival for the patients from the JPN cohort.

	Overall survival			Recurrence-free survival		
Covariate	Hazard ratio	95% Confidence interval	*P*-value	Hazard ratio	95% Confidence interval	*P*-value
iLAS+vs. −	2.61	(1.21, 5.64)	0.014	1.96	(1.14, 3.35)	0.014
Age	1.03	(0.98, 1.08)	0.195	1.04	(1.00, 1.08)	0.033
Gender M vs. F	0.93	(0.36, 2.44)	0.887	0.83	(0.42, 1.67)	0.602
Stage	2.31	(1.49, 3.57)	<0.001	2.16	(1.58, 2.96)	<0.001
Smoking+vs. −	1.08	(0.40, 2.90)	0.880	1.04	(0.51, 2.09)	0.919
Myc high vs. low	0.61	(0.14, 2.63)	0.510	1.14	(0.44, 2.95)	0.789
Mutation+vs. −	0.58	(0.33, 1.01)	0.055	0.62	(0.37, 1.03)	0.066

### Non-randomicity of iLAS

**Figure 5:**
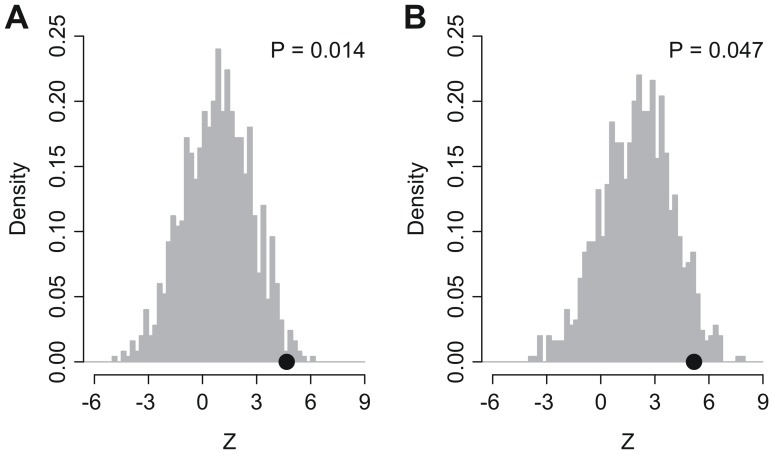
Non-random predictive power of iLAS. *Z* denotes the sum of Cox regression coefficient. The black dots stand for the *Z* values of iLAS. The gray histograms show the distribution of *Z* values for the 1,000 resampled gene signatures with identical size as iLAS under the null hypothesis of no association between iLAS and survival.

A recent study indicates that random gene signatures have a high probability to be associated with survival outcome in breast cancer and published signatures are not significantly more associated with outcome than random predictors [33]. Here, we performed a resampling test to check whether the predictive power of iLAS was by chance or not. We generated 1,000 random gene signatures with identical size as iLAS. Cox proportional hazards regression of survival was conducted for each resampled gene signature. The association between each random gene signature and overall survival was measured by the sum of Cox regression coefficient of the USA2 and JPN cohorts. Similarly, the association between random gene signature and recurrence-free survival was quantified by the sum of Cox regression coefficient of the KOR and JPN cohorts. We found that, for both overall survival and recurrence-free survival, we could reject the null hypothesis that the association between iLAS and survival is by chance (*P* = 0.014 for overall survival and *P* = 0.047 for recurrence-free survival) (Figure 5).

### Differentially Expressed Ion Channel Genes between Lung Adenocarcinoma and Squamous-cell Carcinoma

**Figure 6:**
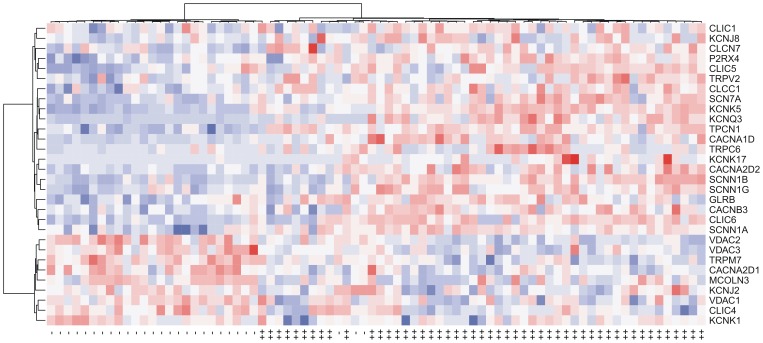
The ion channel genes differentially expressed between adenocarcinoma and squamous-cell carcinoma in the SWE cohort. Each row in the heatmaps is labelled with the corresponding gene symbol. The columns labelled with “++” denote the adenocarcinoma samples while “–” stands for the squamous-cell carcinoma samples. Red represents relatively increased gene expression while blue represents down-regulation.

Squamous-cell carcinoma of the lung is the second-most common type of lung cancer. It accounts for about 30% of all cases of non-small cell lung cancer. We also tested the predictive power of iLAS in squamous-cell carcinoma in the USA2 and KOR datasets, in which the expression data for squamous-cell carcinoma patients were available. There were 53 squamous-cell carcinoma patients with known overall survival data in the USA2 cohort and 75 squamous-cell carcinoma patients with known recurrence-free survival data in the KOR cohort. We didn’t find significant difference in iLAS risk score between adenocarcinoma and squamous-cell carcinoma in both cohorts (*P* = 0.134 and *P* = 0.291 for the USA1 and JPN cohorts, respectively) (Table S8). Kaplan-Meier survival curves indicated that the iLAS signature failed to identify the squamous-cell carcinoma patients with poor clinical outcome in both cohorts (Figure S4), which suggests that there is substantial difference in ion channel gene expression between lung adenocarcinoma and squamous-cell carcinoma. Therefore, we explored the difference in gene expression between the two groups in the SWE cohort, for which the expression data were available for 50 and 28 patients with lung adenocarcinoma and squamous-cell carcinoma, respectively. Two-tailed t-test was used to detect the differentially expressed genes between lung adenocarcinoma and squamous-cell carcinoma. In total, 30 ion channel genes were identified as differentially expressed between the two groups (adjusted *P*<0.05) (Table 4). Twenty-one ion channel genes were up-regulated in adenocarcinoma, while nine ion channel genes were down-regulated (Figure 6).

**Table 4:** Comparison in gene expression level between lung adenocarcinoma and squamous-cell carcinoma.

Gene symbol	Gene title	Fold change^a^	Adjuste*P*-value^b^
CACNA1D	calcium channel, voltage-dependent, L type, alpha 1D subunit	8.02	1.2×10^−8^
CACNA2D1	calcium channel, voltage-dependent, alpha 2/delta subunit 1	0.37	9.1×10^−5^
CACNA2D2	calcium channel, voltage-dependent, alpha 2/delta subunit 2	6.74	8.0×10^−5^
CACNB3	calcium channel, voltage-dependent, beta 3 subunit	2.35	3.4×10^−5^
CLCC1	chloride channel CLIC-like 1	1.51	2.5×10^−5^
CLCN7	chloride channel, voltage-sensitive 7	1.38	6.2×10^−3^
CLIC1	chloride intracellular channel 1	1.13	4.6×10^−2^
CLIC4	chloride intracellular channel 4	0.54	6.0×10^−4^
CLIC5	chloride intracellular channel 5	2.24	1.6×10^−3^
CLIC6	chloride intracellular channel 6	5.10	1.0×10^−6^
GLRB	glycine receptor, beta	1.90	1.9×10^−3^
KCNJ2	potassium inwardly-rectifying channel, subfamily J, member 2	0.77	5.9×10^−3^
KCNJ8	potassium inwardly-rectifying channel, subfamily J, member 8	1.67	2.2×10^−2^
KCNK1	potassium channel, subfamily K, member 1	0.61	1.6×10^−2^
KCNK17	potassium channel, subfamily K, member 17	2.56	1.7×10^−2^
KCNK5	potassium channel, subfamily K, member 5	11.90	8.0×10^−17^
KCNQ3	potassium voltage-gated channel, KQT-like subfamily, member 3	12.53	2.4×10^−11^
MCOLN3	mucolipin 3	0.68	1.3×10^−3^
P2RX4	purinergic receptor P2X, ligand-gated ion channel, 4	1.64	1.9×10^−4^
SCN7A	sodium channel, voltage-gated, type VII, alpha subunit	8.10	1.0×10^−6^
SCNN1A	sodium channel, non-voltage-gated 1 alpha subunit	3.72	9.1×10^−5^
SCNN1B	sodium channel, non-voltage-gated 1, beta subunit	6.68	2.2×10^−7^
SCNN1G	sodium channel, non-voltage-gated 1, gamma subunit	3.28	7.4×10^−4^
TPCN1	two pore segment channel 1	2.61	4.0×10^−9^
TRPC6	transient receptor potential cation channel, subfamily C, member 6	4.24	5.9×10^−3^
TRPM7	transient receptor potential cation channel, subfamily M, member 7	0.54	1.1×10^−6^
TRPV2	transient receptor potential cation channel, subfamily V, member 2	1.41	2.2×10^−2^
VDAC1	voltage-dependent anion channel 1	0.79	3.0×10^−3^
VDAC2	voltage-dependent anion channel 2	0.47	2.9×10^−8^
VDAC3	voltage-dependent anion channel 3	0.47	1.5×10^−4^

a Fold change is calculated by dividing the expression in adenocarcinoma by the expression in squamous-cell carcinoma.

b *P*-value is calculated by two-tailed t-test and adjusted by Benjamini & Hochberg correction.

All the Na^+^ channel genes (*SCN7A*, *SCNN1A*, *SCNN1B*, and *SCNN1G*) listed in Table 4, including one voltage-gated and three non-voltage-gated Na^+^ channels, were all up-regulated in adenocarcinoma. Interestingly, the three genes (*VDAC1*, *VDAC2*, and *VDAC3*,) coding for voltage-dependent anion channels (VDACs), which is a class of porin ion channel located on the outer mitochondrial membrane, were all down-regulated in adenocarcinoma. On the contrary, the expression patterns of Ca^2+^, K^+^, and Cl^−^ channels were more heterogeneous. For example, the directions of the differential expression for *CACNA2D1* and *CACNA2D2* were opposing, as shown in Table 4.

**Figure 7:**
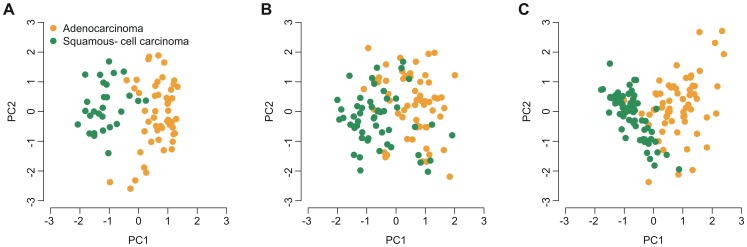
PCA on expression of the ion channel genes differentially expressed between adenocarcinoma and squamous-cell carcinoma. X-axis: the first principal component; Y-axis: the second principal component. Patients with non-small cell lung cancer from the (A) SWE, (B) USA2, and (C) KOR cohorts were considered here.

We validated the ion channel genes that were differentially expressed between adenocarcinoma and squamous-cell carcinoma. We looked into two independent lung cancer datasets (USA2 and KOR) where gene expression data were available for both adenocarcinoma and squamous-cell carcinoma patients. We observed a significant difference (adjusted *P*<0.05) between the two groups in at least one validation cohort for each gene, except for *CLIC4*, *KCNK1*, and *MCOLN3* (Table S9). PCA indicated that patients with adenocarcinoma can be well distinguished from those with squamous-cell carcinoma not only in the SWE cohort (Figure 7A), but also in the USA2 (Figure 7B) and KOR (Figure 7C) cohorts, based only on the expression of the 30 differentially expressed ion channel genes (Figure 7). Again, a significant positive correlation in log10-transformed *P*-value (generated by two-tailed t-test) was observed between the SWE and USA2 cohorts (Figure S5A) and between the SWE and KOR cohorts (Figure S5B). The direction of differential expression in the SWE cohort was reproduced in the USA1 and KOR cohorts (Figures S5C and S5D). Unsupervised hierarchical cluster analysis also demonstrated a very similar expression pattern of the 30 differentially expressed ion channel genes among the SWE, USA2 and KOR cohorts (Figures S6 and S7).

## Discussion

Ion channels have been implicated in the regulation of a variety of biological and physiological processes ranging from cellular secretion to electrical signaling. Because of the ubiquitous and critical involvement of ion channels in diverse biological functions, dysfunction of ion channels has been attributed to multiple human diseases [34], including cancers. It is now known that ion channels regulate the cancer processes at all stages by affecting cell-cycle progression and proliferation, apoptosis, cancer cell metabolism and tumor microenvironment, and tumor cell motility and invasion [35]. However, the roles of ion channels in cancer pathology are as diverse as the ion channel families themselves. It is currently difficult to assign a detailed mechanism for each ion channel in the proliferation, invasion, and metastasis of tumor cells. Therefore, a comprehensive investigation of all the channels and their possible functions in cancer progression is beyond the scope of this study. Here, we systematically examined the ion channel gene expression profiling in lung adenocarcinoma.

First, we compared the expression of ion channel genes between normal and tumor tissues in patients with lung adenocarcinoma. Thirty-seven ion channel genes (iLAS) were identified as being differentially expressed between the two groups, which was largely reproduced in the validation dataset. The expression patterns of K^+^ channel and Cl^−^ channels were heterogeneous. Either up- or down-regulation in tumor tissues was found for K^+^ and Cl^−^ channels in this study, which is consistent with the observations in breast cancer (unpublished data). On the contrary, genes coding for the α subunit of voltage-dependent Ca^2+^ channels, Na^+^ channels, and the polycystin and mucolipin subfamilies of TRP channels were all found to be down-regulated in tumor tissues. Dysregulation of Ca^2+^ channels is known to contribute significantly to cell proliferation in cancers [36]. Up-regulation of voltage-gated Ca^2+^ channels has been observed in colon cancer cells [6] and small cell lung cancers [36]. However, the α subunits of voltage-dependent Ca^2+^ channels listed in Table 1 were all down-regulated in tumor tissues of lung adenocarcinoma, which is in accordance with findings in breast cancer. It was found that voltage-gated Ca^2+^ ion channel genes are down-regulated in p53 mutant tumors and/or tumors with higher histological grade in breast cancer (unpublished data). Increased expression of voltage-gated Na^+^ channels has been reported in many cancer cell lines, including small/non-small cell lung cancers [11], [37]. However, we presented the opposite direction of differential expression here, which may be due to differences between cell lines and primary tumors. TRP channels have been shown to have a significant effect on a variety of pathological processes [38], [39]. The role of TRP channels in human cancer, with respect to enhanced proliferation, aberrant differentiation, and tumor invasion, has been increasingly clarified [38], [40], [41], [42]. Either increased or decreased expression in tumor tissues compared to normal controls of TRP channels has been observed depending on the type or stage of the cancer [43], [44], [45], [46]. Although the polycystin and mucolipin subfamilies of TRP channels are all down-regulated in tumor tissues, we also found one up-regulated TRP channel gene (*TRPM2*) in this study.

Next, we tested the prognostic power of ion channel genes in lung adenocarcinoma. A risk score was assigned to each lung adenocarcinoma patient based on the expression of iLAS. We demonstrated that iLAS is a promising prognostic molecular signature. iLAS risk score effectively predicts both overall survival and recurrence-free survival in several independent cohorts from different regions of the world. The most common cause of lung cancer is long-term tobacco smoking. Unsurprisingly, we found that the iLAS scores for ever-smokers were higher than those for never-smokers. However, multivariate analysis confirmed that iLAS remained a significant predictive factor for survival in relation to age, gender, stage, smoking history, Myc level, and *EGFR*/*KRAS*/*ALK* gene mutation status, which suggest that iLAS is largely independent of the traditional clinical and pathological factors.

Finally, we compared the ion channel gene expression pattern between lung adenocarcinoma and squamous-cell carcinoma. Thirty ion channel genes were found to be dysregulated between the two groups. It is interesting that all three VDAC genes were down-regulated in adenocarcinoma. Usually, VDACs are located in the outer mitochondrial membrane [47]. It was reported that higher VDAC1 expression level predicted poor outcome in non-small cell lung cancer [48]. However, our results indicate that there is substantial difference in VDAC gene expression within the subtypes of non-small cell lung cancer. The differentially expressed ion channel genes between adenocarcinoma and squamous-cell carcinoma will be useful to improve the histo-pathological classification of non-small cell lung cancer. Misdiagnosis may be prevented and corrected according to the molecularly defined subtypes.

In summary, we investigated the gene expression profile of ion channels in lung adenocarcinoma. We identified a molecular signature iLAS, which represents a promising prognostic biomarker in lung adenocarcinoma. When working cooperatively with known clinicopathological factors, iLAS may enhance prediction accuracy in identifying patients at higher risk for recurrence and death. Also, we suggest that classification and diagnosis of non-small cell lung cancer can be potentially improved by ion channel gene expression pattern. Although there is a long way from treating cancer as a channelopathy, ion channels are potential new targets for therapy in human cancers.

## Supporting Information

Figure S1 **Distribution of risk score.** The red dash lines indicate the median of risk score. There is no significant deviation between zero and the median of risk score in each cohort.
(PDF)

Figure S2 **Validation for the ion channel genes differentially expressed between normal and tumor tissues.** The ion channel genes differentially expressed between normal and tumor tissues in the TWN cohort were validated in the USA1 cohort. Paired normal and tumor tissues from 33 lung adenocarcinoma patients were included in the comparison. In total, 23 ion channel genes were identified as dysregulated in the USA1 cohort. Y-axis: log2-transformed expression values.
(PDF)

Figure S3 **Coefficient of Cox proportional hazards regression.** Cox hazards regression was conducted for each gene in iLAS. Each dot denotes one iLAS gene. A significant positive correlation (*P*<0.05) was identified between fold change (tumor/normal) and Cox regression coefficient for each dataset except the KOR cohort.
(PDF)

Figure S4 **Kaplan-Meier curves for the patients with squamous-cell lung carcinoma.** Red curves are for the iLAS positive patients while blue curves are for the iLAS negative patients. iLAS positive patients were defined as those having a iLAS risk score greater than the group median score. *P*-values were calculated by log-rank tests for the differences in survival between the iLAS positive and negative groups. (A) iLAS failed to predict overall survival in the USA2 cohort; (B) iLAS failed to predict recurrence-free survival in the KOR cohort.
(PDF)

Figure S5 **Comparison between the SWE, USA2 and KOR cohorts.** (A) Correlation of *P*-value generated by t-test (adenocarcinoma vs. squamous-cell carcinoma) between the SWE and USA2 cohorts; (B) Correlation of *P*-value generated by t-test (adenocarcinoma vs. squamous-cell carcinoma) between the SWE and KOR cohorts; (C) Correlation of fold change of gene expression level (adenocarcinoma vs. squamous-cell carcinoma) between the SWE and USA2 cohorts; and (D) Correlation of fold change of gene expression level (adenocarcinoma vs. squamous-cell carcinoma) between the SWE and KOR cohorts.
(PDF)

Figure S6 **Validation in the USA2 cohort for the genes differentially expressed between adenocarcinoma and squamous-cell carcinoma.** The differentially expressed ion channel genes were derived from the SWE cohort. Each row in the heatmaps is labelled with the corresponding gene symbol. The columns labelled with “++” denote the adenocarcinoma samples while “–” stands for the squamous-cell carcinoma samples. Red represents relatively increased gene expression while blue represents down-regulation.
(PDF)

Figure S7 **Validation in the KOR cohort for the genes differentially expressed between adenocarcinoma and squamous-cell carcinoma.** The differentially expressed ion channel genes were derived from the SWE cohort. Each row in the heatmaps is labelled with the corresponding gene symbol. The columns labelled with “++” denote the adenocarcinoma samples while “–” stands for the squamous-cell carcinoma samples. Red represents relatively increased gene expression while blue represents down-regulation.
(PDF)

Table S1 **Gene expression datasets of lung cancer from GEO database.**
(PDF)

Table S2 **Ion channel genes involved in this study.**
(PDF)

Table S3 **Comparison between the TWN and USA1 cohorts.**
(PDF)

Table S4 **Validation for the difference in gene expression level between normal and tumor tissues in the USA1 cohort.**
(PDF)

Table S5 **Univariate Cox proportional hazards regression of survival by iLAS continuous score for the lung adenocarcinoma patients.**
(PDF)

Table S6 **Means, medians, and standard deviations of iLAS risk score for the patients with and without smoking history in the USA2 and JPN cohorts.**
(PDF)

Table S7 **Multivariate Cox proportional hazard regression of survival for the patients from the JPN cohort.**
(PDF)

Table S8 **Means, medians, and standard deviations of iLAS risk score for the adenocarcinoma and squamous-cell carcinoma patients in the USA2 and KOR cohorts.**
(PDF)

Table S9 **Validation for the difference in gene expression level between adenocarcinoma and squamous-cell carcinoma in the USA2 and KOR cohorts.**
(PDF)
