# Supplementary figures and images for: Ion Channel Gene Expression in Lung Adenocarcinoma: Potential Role in Prognosis and Diagnosis
Source: PLoS One. 2014 Jan 23;9(1):e86569. doi: 10.1371/journal.pone.0086569 (PMC3900557; doi:10.1371/journal.pone.0086569)

**USA2**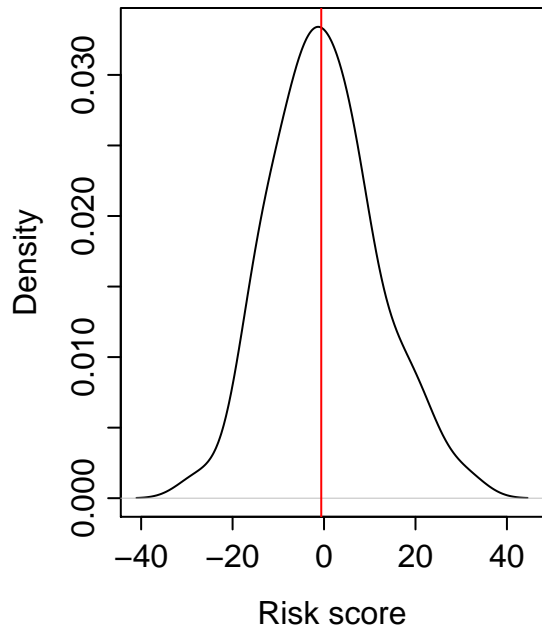**JPN**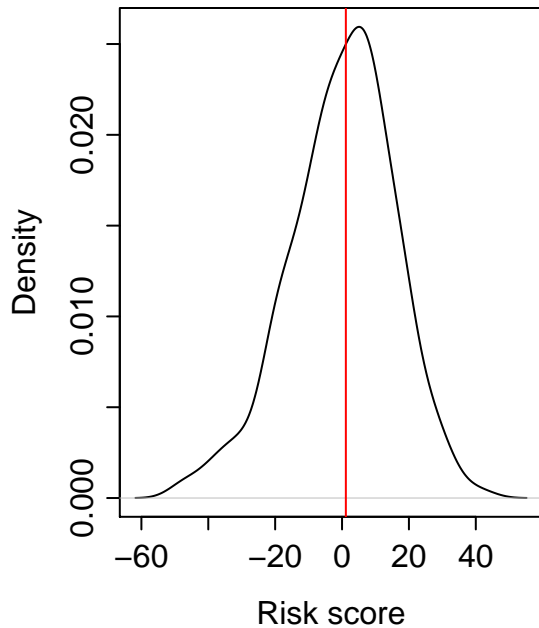**KOR**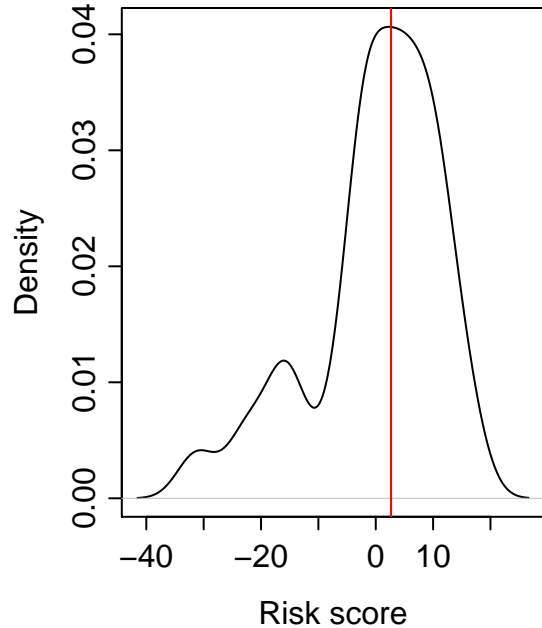

Supplement: Figure S1 — Distribution of risk score. The red dash lines indicate the median of risk score. There is no significant deviation between zero and the median of risk score in each cohort. (PDF) [file pone.0086569.s001.pdf]

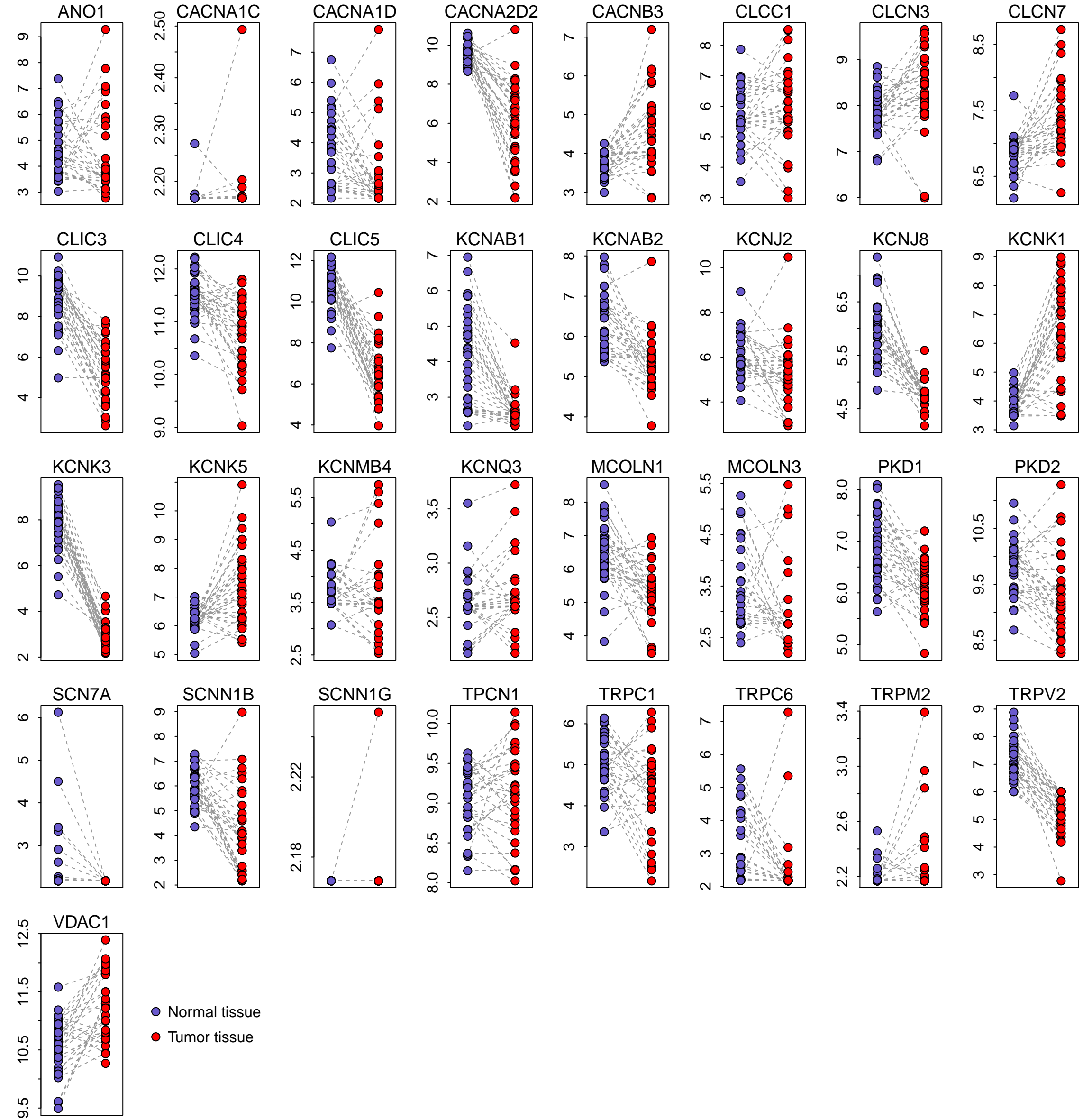

Supplement: Figure S2 — Validation for the ion channel genes differentially expressed between normal and tumor tissues. The ion channel genes differentially expressed between normal and tumor tissues in the TWN cohort were validated in the USA1 cohort. Paired normal and tumor tissues from 33 lung adenocarcinoma patients were included in the comparison. In total, 23 ion channel genes were identified as dysregulated in the USA1 cohort. Y-axis: log2-transformed expression values. (PDF) [file pone.0086569.s002.pdf]

**A**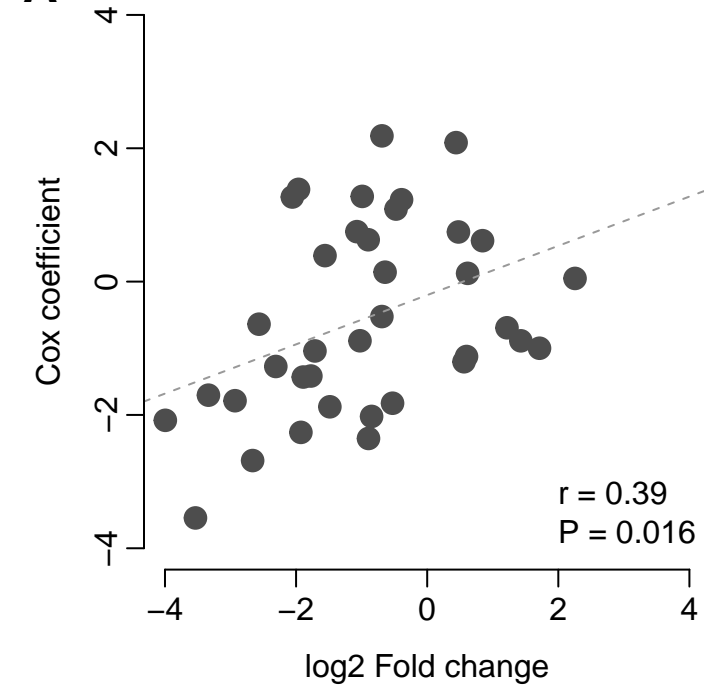**B**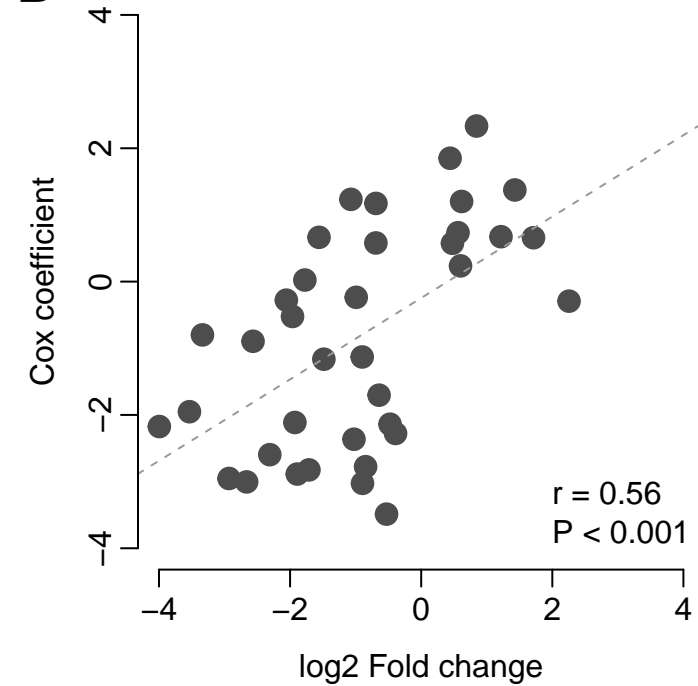**C**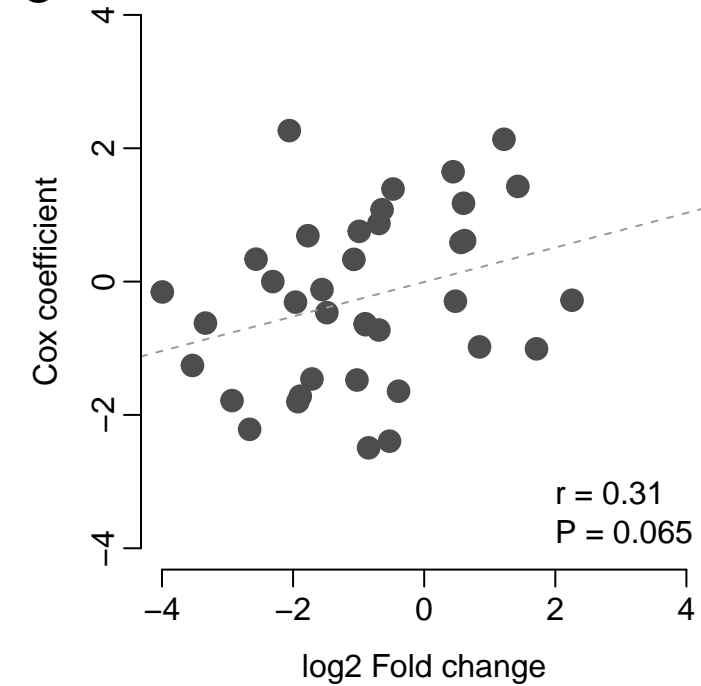**D**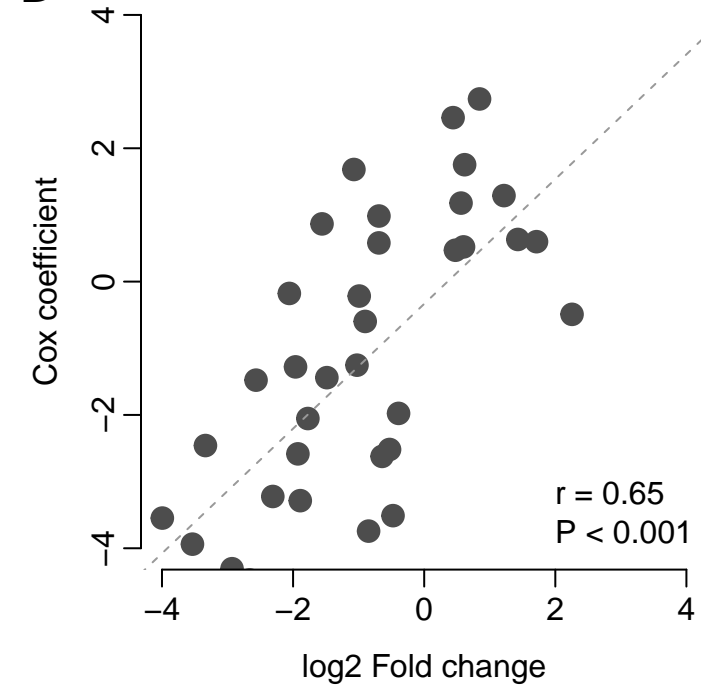

Supplement: Figure S3 — Coefficient of Cox proportional hazards regression. Cox hazards regression was conducted for each gene in iLAS. Each dot denotes one iLAS gene. A significant positive correlation (P<0.05) was identified between fold change (tumor/normal) and Cox regression coefficient for each dataset except the KOR cohort. (PDF) [file pone.0086569.s003.pdf]

**A**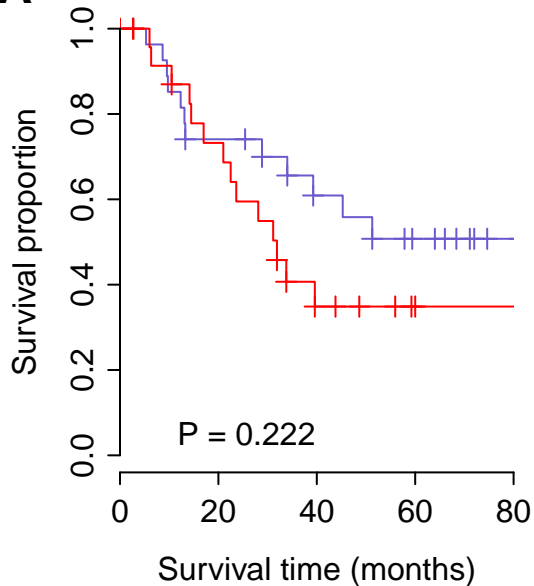**B**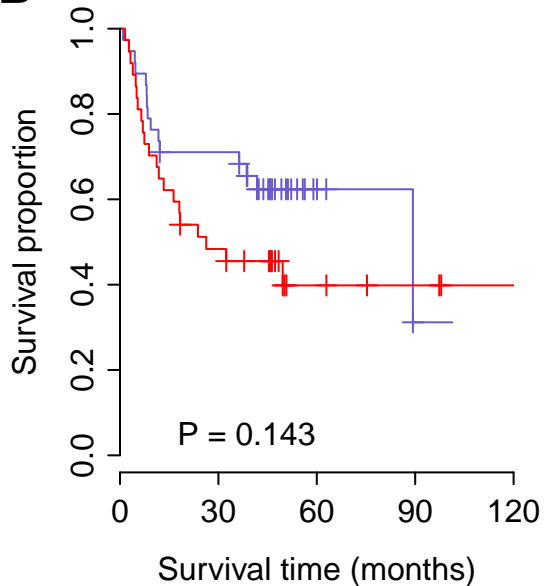

Supplement: Figure S4 — Kaplan-Meier curves for the patients with squamous-cell lung carcinoma. Red curves are for the iLAS positive patients while blue curves are for the iLAS negative patients. iLAS positive patients were defined as those having a iLAS risk score greater than the group median score. P-values were calculated by log-rank tests for the differences in survival between the iLAS positive and negative groups. (A) iLAS failed to predict overall survival in the USA2 cohort; (B) iLAS failed to predict recurrence-free survival in the KOR cohort. (PDF) [file pone.0086569.s004.pdf]

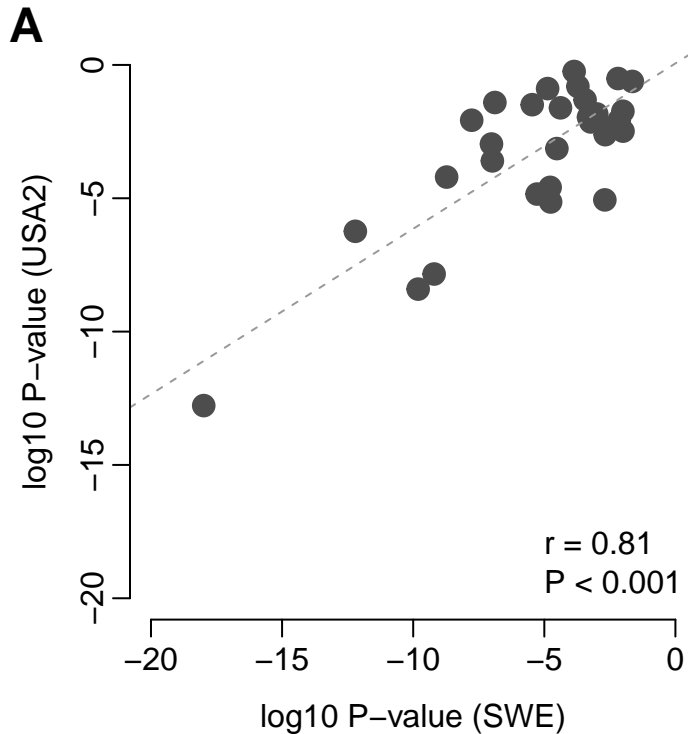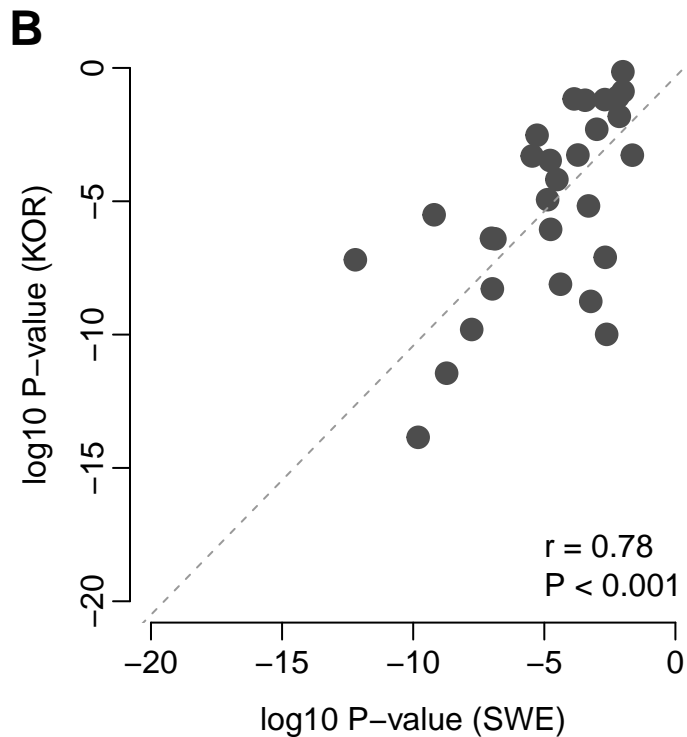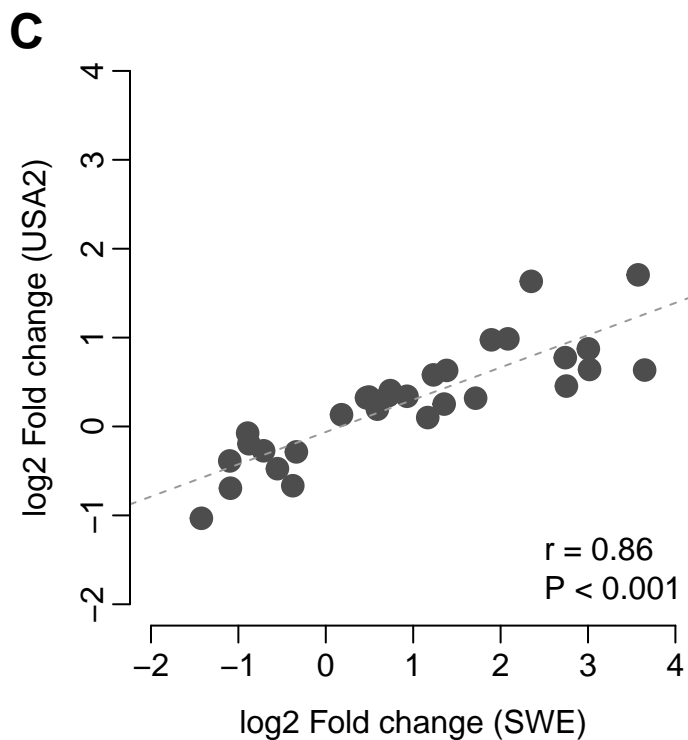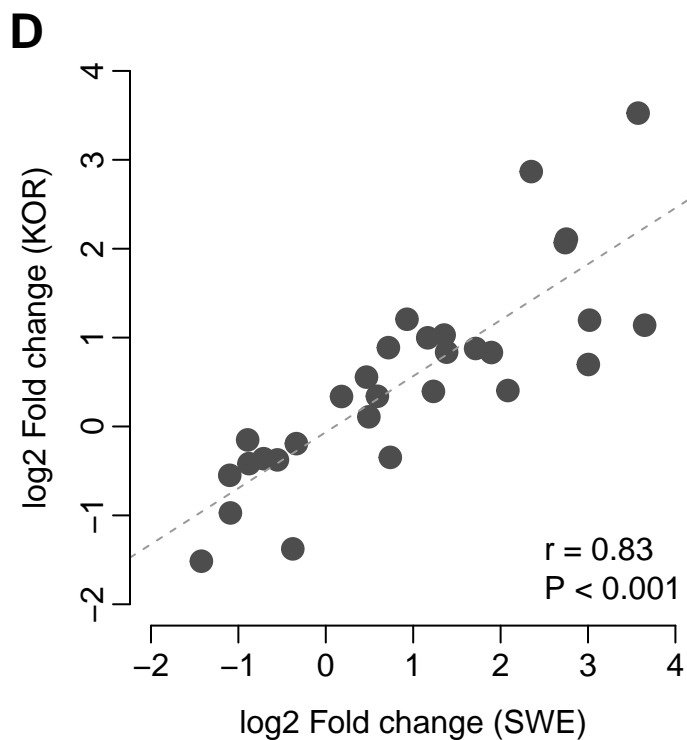

Supplement: Figure S5 — Comparison between the SWE, USA2 and KOR cohorts. (A) Correlation of P-value generated by t-test (adenocarcinoma vs. squamous-cell carcinoma) between the SWE and USA2 cohorts; (B) Correlation of P-value generated by t-test (adenocarcinoma vs. squamous-cell carcinoma) between the SWE and KOR cohorts; (C) Correlation of fold change of gene expression level (adenocarcinoma vs. squamous-cell carcinoma) between the SWE and USA2 cohorts; and (D) Correlation of fold change of gene expression level (adenocarcinoma vs. squamous-cell carcinoma) between the SWE and KOR cohorts. (PDF) [file pone.0086569.s005.pdf]

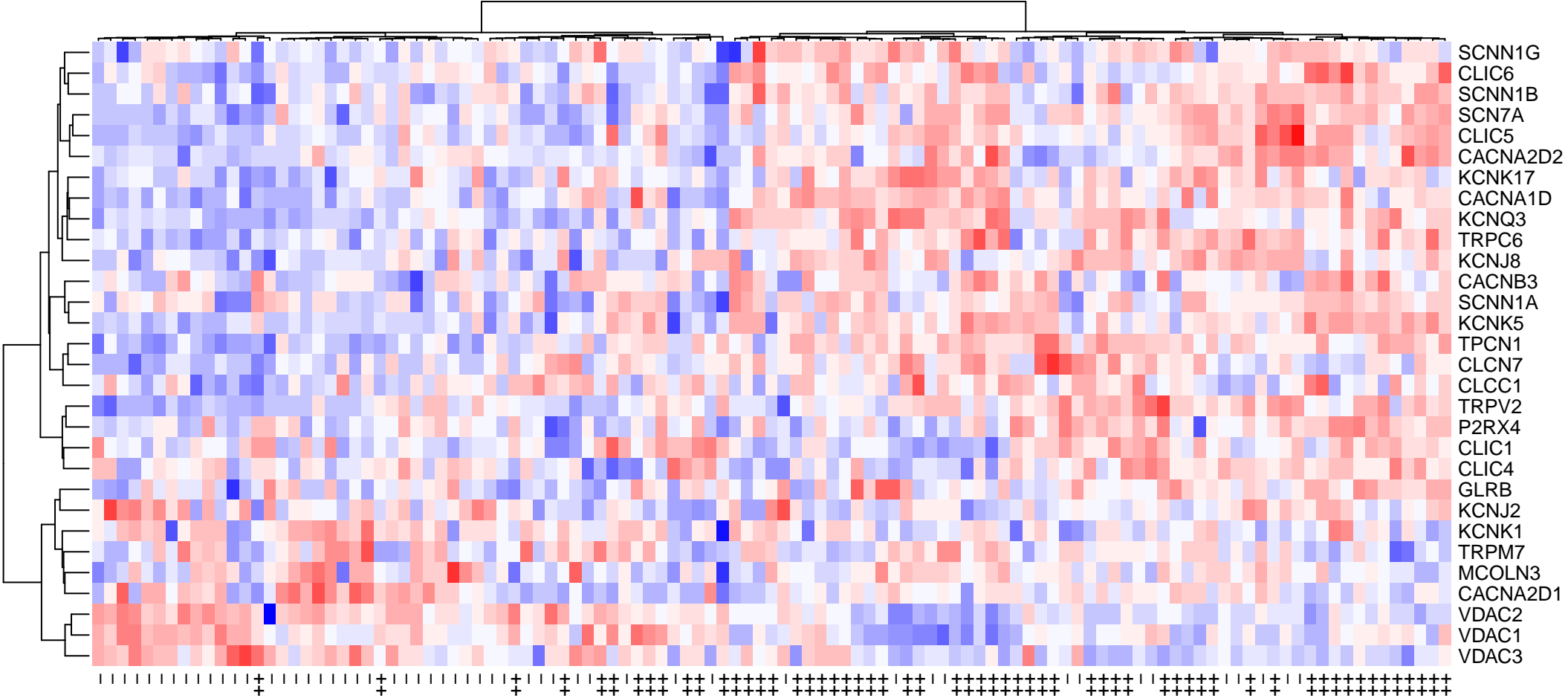

Supplement: Figure S6 — Validation in the USA2 cohort for the genes differentially expressed between adenocarcinoma and squamous-cell carcinoma. The differentially expressed ion channel genes were derived from the SWE cohort. Each row in the heatmaps is labelled with the corresponding gene symbol. The columns labelled with “++” denote the adenocarcinoma samples while “–” stands for the squamous-cell carcinoma samples. Red represents relatively increased gene expression while blue represents down-regulation. (PDF) [file pone.0086569.s006.pdf]

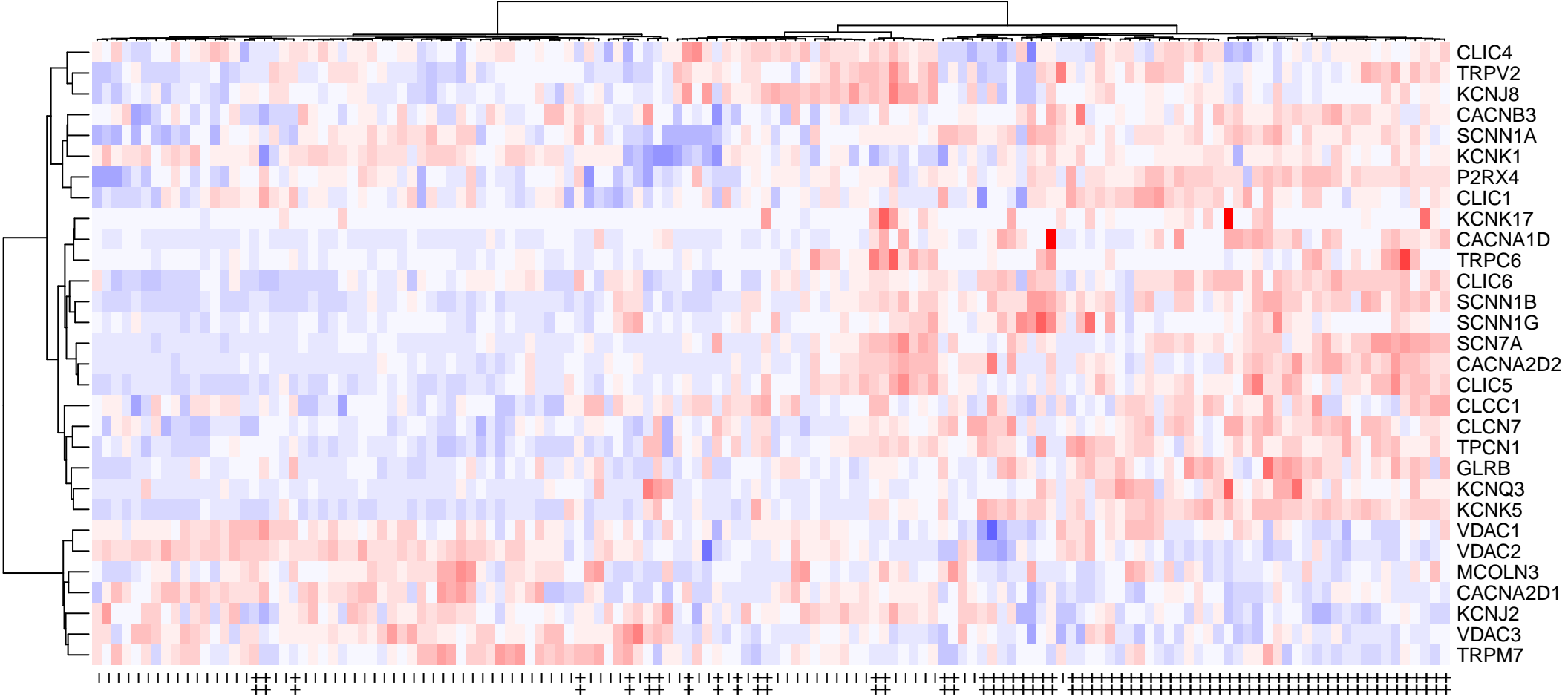

Supplement: Figure S7 — Validation in the KOR cohort for the genes differentially expressed between adenocarcinoma and squamous-cell carcinoma. The differentially expressed ion channel genes were derived from the SWE cohort. Each row in the heatmaps is labelled with the corresponding gene symbol. The columns labelled with “++” denote the adenocarcinoma samples while “–” stands for the squamous-cell carcinoma samples. Red represents relatively increased gene expression while blue represents down-regulation. (PDF) [file pone.0086569.s007.pdf]
