# Supplementary material for: Ion Channel Gene Expression in Lung Adenocarcinoma: Potential Role in Prognosis and Diagnosis
Source: PLoS One. 2014 Jan 23;9(1):e86569. doi: 10.1371/journal.pone.0086569 (PMC3900557; doi:10.1371/journal.pone.0086569)
Supplement: Table S1 — Gene expression datasets of lung cancer from GEO database. (PDF) [file pone.0086569.s008.pdf]

Table S1. Gene expression datasets of lung cancer from GEO database

| Organization of data source | Abbreviation | GEO accession | Platform | Samples size [a] | Paired |
|---|---|---|---|---|---|
| National Cancer Center Research Institute, Japan | JPN | GSE31210 | Affymetrix Human Genome U133 Plus 2.0 | A: 226 | No |
| Samsung Medical Center, Korea | KOR | GSE8894 | Affymetrix Human Genome U133 Plus 2.0 | A: 63; S: 75 | No |
| Uppsala Array Platform, Sweden | SWE | GSE28571 | Affymetrix Human Genome U133 Plus 2.0 | A: 50; S: 28 | No |
| National Taiwan University, Taiwan | TWN | GSE19804 | Affymetrix Human Genome U133 Plus 2.0 | A: 56; N: 56 | Yes |
| National Cancer Institute, United States | USA1 | GSE10072 | Affymetrix Human Genome U133A | A: 33; N: 33 | Yes |
| Duke University, United States | USA2 | GSE3141 | Affymetrix Human Genome U133 Plus 2.0 | A: 58; S: 53 | No |

[a] Only the samples used in this study were counted. A: lung adenocarcinoma tumor sample; S: squamous-cell lung carcinoma tumor sample; N: paired normal sample.
