# Supplementary material for: Ion Channel Gene Expression in Lung Adenocarcinoma: Potential Role in Prognosis and Diagnosis
Source: PLoS One. 2014 Jan 23;9(1):e86569. doi: 10.1371/journal.pone.0086569 (PMC3900557; doi:10.1371/journal.pone.0086569)
Supplement: Table S3 — Comparison between the TWN and USA1 cohorts. (PDF) [file pone.0086569.s010.pdf]

Table S3. Comparison between the TWN and USA1 cohorts

	TWN	USA1	*P*-value
Age (mean ± standard deviation)	60.9 ± 10.3	65.1 ± 8.5	0.042 by t-test
Gender (male/female)	0/56	22/11	< 0.001 by χ^2 test
Never-smoker/ever-smoker	56/0	11/22	< 0.001 by χ^2 test
