# Supplementary material for: Ion Channel Gene Expression in Lung Adenocarcinoma: Potential Role in Prognosis and Diagnosis
Source: PLoS One. 2014 Jan 23;9(1):e86569. doi: 10.1371/journal.pone.0086569 (PMC3900557; doi:10.1371/journal.pone.0086569)
Supplement: Table S4 — Validation for the difference in gene expression level between normal and tumor tissues in the USA1 cohort. (PDF) [file pone.0086569.s011.pdf]

Table S4. Validation for the difference in gene expression level between normal and tumor tissues in the USA1 cohort

Gene symbol	Gene title	Fold change[a]	Adjusted *P*-value[b]
ANO1	anoctamin 1, calcium activated chloride channel	0.92	7.3E-01
CACNA1C	calcium channel, voltage-dependent, L type, alpha 1C subunit	1.01	4.6E-01
CACNA1D	calcium channel, voltage-dependent, L type, alpha 1D subunit	0.57	7.3E-03
CACNA2D2	calcium channel, voltage-dependent, alpha 2/delta subunit 2	0.08	9.2E-11
CACNB3	calcium channel, voltage-dependent, beta 3 subunit	1.64	5.5E-04
CLCC1	chloride channel CLIC-like 1	1.16	3.8E-01
CLCN3	chloride channel, voltage-sensitive 3	1.37	4.8E-03
CLCN7	chloride channel, voltage-sensitive 7	1.41	6.4E-05
CLIC3	chloride intracellular channel 3	0.08	2.9E-12
CLIC4	chloride intracellular channel 4	0.62	1.8E-05
CLIC5	chloride intracellular channel 5	0.05	6.2E-13
KCNAB1	potassium voltage-gated channel, shaker-related subfamily, beta member 1	0.37	3.6E-07
KCNAB2	potassium voltage-gated channel, shaker-related subfamily, beta member 2	0.49	2.9E-07
KCNJ2	potassium inwardly-rectifying channel, subfamily J, member 2	0.55	6.5E-03
KCNJ8	potassium inwardly-rectifying channel, subfamily J, member 8	0.43	4.5E-11
KCNK1	potassium channel, subfamily K, member 1	5.29	4.2E-08
KCNK3	potassium channel, subfamily K, member 3	0.03	1.2E-20
KCNK5	potassium channel, subfamily K, member 5	2.12	2.9E-04
KCNMB4	potassium large conductance calcium-activated channel, subfamily M, beta member 4	1.00	9.7E-01
KCNQ3	potassium voltage-gated channel, KQT-like subfamily, member 3	1.06	1.4E-01
MCOLN1	mucolipin 1	0.45	1.8E-05
MCOLN3	mucolipin 3	0.78	9.4E-02
PKD1	polycystic kidney disease 1 (autosomal dominant)	0.59	2.2E-06
PKD2	polycystic kidney disease 2 (autosomal dominant)	0.76	3.4E-03
SCN7A	sodium channel, voltage-gated, type VII, alpha subunit	0.81	5.5E-02
SCNN1B	sodium channel, non-voltage-gated 1, beta subunit	0.26	3.0E-06
SCNN1G	sodium channel, non-voltage-gated 1, gamma subunit	1.00	3.8E-01
TPCN1	two pore segment channel 1	1.05	4.7E-01
TRPC1	transient receptor potential cation channel, subfamily C, member 1	0.64	4.7E-03
TRPC6	transient receptor potential cation channel, subfamily C, member 6	0.63	2.8E-02
TRPM2	transient receptor potential cation channel, subfamily M, member 2	1.06	1.2E-01
TRPV2	transient receptor potential cation channel, subfamily V, member 2	0.27	5.4E-12
VDAC1	voltage-dependent anion channel 1	1.54	2.8E-05

[a] Fold change is calculated by dividing the expression of tumor tissue by the expression of normal tissue.

[b] *P*-value is calculated by paired t-test and adjusted by Benjamini & Hochberg correction.
