# Supplementary material for: Ion Channel Gene Expression in Lung Adenocarcinoma: Potential Role in Prognosis and Diagnosis
Source: PLoS One. 2014 Jan 23;9(1):e86569. doi: 10.1371/journal.pone.0086569 (PMC3900557; doi:10.1371/journal.pone.0086569)
Supplement: Table S5 — Univariate Cox proportional hazards regression of survival by iLAS continuous score for the lung adenocarcinoma patients. (PDF) [file pone.0086569.s012.pdf]

Table S5. Univariate Cox proportional hazards regression of survival by iLAS continuous score for the lung adenocarcinoma patients

Gategory	Cohort	Number of patients	Hazard ratio	95% Confidence interval	*P*-value
Overall survival	USA2	58	1.02	(0.99, 1.06)	0.142
	JPN	226	1.04	(1.01, 1.06)	0.002
Recurrence-free survival	KOR	63	1.03	(1.00, 1.07)	0.087
	JPN	226	1.04	(1.02, 1.06)	< 0.001
