# Supplementary material for: Ion Channel Gene Expression in Lung Adenocarcinoma: Potential Role in Prognosis and Diagnosis
Source: PLoS One. 2014 Jan 23;9(1):e86569. doi: 10.1371/journal.pone.0086569 (PMC3900557; doi:10.1371/journal.pone.0086569)
Supplement: Table S6 — Means, medians, and standard deviations of iLAS risk score for the patients with and without smoking history in the USA2 and JPN cohorts. (PDF) [file pone.0086569.s013.pdf]

Table S6. Means, medians, and standard deviations of iLAS risk score for the patients with and without smoking history in the USA2 and JPN cohorts

	USA2		JPN	
	Never-smoker	Ever-smoker	Never-smoker	Ever-smoker
Mean	-3.46	1.69	-2.92	3.03
Median	-2.31	0.23	-2.05	4.05
Standard deviation	9.11	8.52	15.58	15.28
