# Supplementary material for: Ion Channel Gene Expression in Lung Adenocarcinoma: Potential Role in Prognosis and Diagnosis
Source: PLoS One. 2014 Jan 23;9(1):e86569. doi: 10.1371/journal.pone.0086569 (PMC3900557; doi:10.1371/journal.pone.0086569)
Supplement: Table S7 — Multivariate Cox proportional hazard regression of survival for the patients from the JPN cohort. (PDF) [file pone.0086569.s014.pdf]

Table S7. Multivariate Cox proportional hazard regression of survival for the patients from the JPN cohort

	Overall survival			Recurrence-free survival		
Covariate	Hazard ratio	95% Confidence interval	*P*-value	Hazard ratio	95% Confidence interval	*P*-value
iLAS [a]	1.02	(1.00, 1.05)	0.017	1.03	(1.01, 1.05)	< 0.001
Age	1.03	(0.98, 1.08)	0.291	1.03	(1.00, 1.07)	0.067
Gender M vs.F	0.83	(0.32, 2.12)	0.693	0.77	(0.38, 1.53)	0.454
Stage	2.22	(1.44, 3.43)	< 0.001	2.06	(1.50, 2.82)	< 0.001
Smoking + vs. -	1.10	(0.42, 2.86)	0.844	0.97	(0.48, 1.95)	0.927
Myc high vs. low	0.61	(0.14, 2.62)	0.506	1.06	(0.41, 2.74)	0.904
Mutation + vs. -	0.50	(0.25, 1.00)	0.049	0.59	(0.35, 0.99)	0.043

[a] iLAS status was treated as continuous variable.
