# Supplementary material for: Ion Channel Gene Expression in Lung Adenocarcinoma: Potential Role in Prognosis and Diagnosis
Source: PLoS One. 2014 Jan 23;9(1):e86569. doi: 10.1371/journal.pone.0086569 (PMC3900557; doi:10.1371/journal.pone.0086569)
Supplement: Table S8 — Means, medians, and standard deviations of iLAS risk score for the adenocarcinoma and squamous-cell carcinoma patients in the USA2 and KOR cohorts. (PDF) [file pone.0086569.s015.pdf]

Table S8. Means, medians, and standard deviations of iLAS risk score for the adenocarcinoma and squamous-cell carcinoma patients in the USA2 and KOR cohorts

	USA2		KOR	
	adenocarcinoma	squamous-cell carcinoma	adenocarcinoma	squamous-cell carcinoma
Mean	-1.69	1.85	-1.19	1.00
Median	-2.06	4.74	1.13	4.38
Standard deviation	10.90	13.50	11.46	12.84
