# Supplementary material for: Ion Channel Gene Expression in Lung Adenocarcinoma: Potential Role in Prognosis and Diagnosis
Source: PLoS One. 2014 Jan 23;9(1):e86569. doi: 10.1371/journal.pone.0086569 (PMC3900557; doi:10.1371/journal.pone.0086569)
Supplement: Table S9 — Validation for the difference in gene expression level between adenocarcinoma and squamous-cell carcinoma in the USA2 and KOR cohorts. (PDF) [file pone.0086569.s016.pdf]

Table S9. Validation for the difference in gene expression level between adenocarcinoma and squamous-cell carcinoma in the USA2 and KOR cohorts

	USA2		KOR	
Gene symbol	Fold change[a]	Adjusted *P*-value[b]	Fold change[a]	Adjusted *P*-value[b]
CACNA1D	1.83	1.4E-07	1.62	6.7E-06
CACNA2D1	0.49	4.3E-05	0.35	2.0E-06
CACNA2D2	1.37	1.5E-01	4.31	2.1E-05
CACNB3	1.50	6.2E-05	1.31	4.1E-03
CLCC1	1.14	4.2E-02	1.27	7.7E-04
CLCN7	1.25	8.4E-03	1.47	7.7E-10
CLIC1	1.10	2.6E-01	1.26	7.7E-04
CLIC4	0.95	5.7E-01	0.90	7.6E-02
CLIC5	1.07	1.7E-02	2.00	1.3E-05
CLIC6	3.10	7.6E-04	7.30	2.2E-08
GLRB	1.27	1.4E-02	2.31	8.8E-09
KCNJ2	0.63	5.6E-03	0.39	2.4E-07
KCNJ8	1.32	2.6E-02	0.79	7.2E-01
KCNK1	0.83	3.2E-01	0.78	8.8E-02
KCNK17	1.19	1.4E-02	2.04	1.9E-02
KCNK5	3.26	5.0E-12	11.52	6.4E-21
KCNQ3	1.55	4.3E-06	2.20	2.1E-07
MCOLN3	0.72	5.9E-02	0.77	7.4E-02
P2RX4	1.27	3.4E-02	1.85	2.9E-08
SCN7A	1.56	2.7E-03	2.29	1.0E-06
SCNN1A	1.97	9.6E-05	1.78	5.6E-04
SCNN1B	1.71	1.4E-02	4.19	9.3E-10
SCNN1G	1.25	1.7E-01	1.84	7.7E-04
TPCN1	1.55	5.9E-08	1.78	2.1E-13
TRPC6	1.98	4.3E-05	1.32	7.5E-02
TRPM7	0.87	4.9E-02	0.75	1.0E-06
TRPV2	1.26	7.0E-03	1.08	1.4E-01
VDAC1	0.82	2.2E-02	0.87	6.5E-03
VDAC2	0.62	2.1E-04	0.51	3.6E-11
VDAC3	0.76	2.0E-03	0.68	1.2E-04

[a] Fold change is calculated by dividing the expression in adenocarcinoma by the expression in squamous-cell carcinoma.

[b] *P*-value is calculated by two-tailed t-test and adjusted by Benjamini & Hochberg correction.
